# Cerebellar lncRNA Expression Profile Analysis of SCA3/MJD Mice

**DOI:** 10.1155/2018/5383517

**Published:** 2018-06-25

**Authors:** Zhe Long, Tianjiao Li, Zhao Chen, Yun Peng, Chunrong Wang, Xiaocan Hou, Hongyu Yuan, Puzhi Wang, Yue Xie, Lang He, Xin Zhou, Huirong Peng, Rong Qiu, Kun Xia, Beisha Tang, Hong Jiang

**Affiliations:** ^1^Department of Neurology, Xiangya Hospital, Central South University, Changsha, Hunan 410008, China; ^2^Sydney Medical School and the Brain & Mind Institute, The University of Sydney, 94 Mallett St, Camperdown, NSW 2050, Australia; ^3^School of Information Science and Engineering, Central South University, Changsha, Hunan 410083, China; ^4^Laboratory of Medical Genetics, Central South University, Changsha, Hunan 410078, China; ^5^National Clinical Research Center for Geriatric Diseases, Xiangya Hospital, Central South University, Changsha, Hunan 410008, China; ^6^Key Laboratory of Hunan Province in Neurodegenerative Disorders, Central South University, Changsha, Hunan 410008, China; ^7^Parkinson's Disease Center of Beijing Institute for Brain Disorders, Beijing 100069, China; ^8^Collaborative Innovation Center for Brain Science, Shanghai 200032, China; ^9^Collaborative Innovation Center for Genetics and Development, Shanghai 200433, China; ^10^Xinjiang Medical University, Xinjiang 830011, China

## Abstract

Spinocerebellar ataxia type 3 (SCA3) or Machado-Joseph disease (MJD) is the most common autosomal dominant spinocerebellar ataxia in China with highly clinical heterogeneity, such as progressive cerebellar ataxia, dysarthria, pyramidal signs, external ophthalmoplegia, dysphagia, and distal muscle atrophy. It is caused by the abnormal expansion of CAG repeats in a coding region of *ATXN3*. However, by focusing on the *ATXN3* itself cannot fully explain the heterogeneous clinical features of SCA3/MJD. With the discovery of the increasing number of long noncoding RNAs (lncRNAs) that are believed to be involved in spinocerebellar ataxia type 8 (SCA8) and Huntington disease (HD), we wonder whether the lncRNAs are differentially expressed in the SCA3/MJD patients compared to the nonpatients. As the first step, we used lncRNA-Seq to investigate differential expression of the lncRNAs in the SCA3/MJD mice. Two known lncRNAs, n297609 and n297477, and a novel lncRNA TCONS_00072962 have been identified in SCA3/MJD mice with abnormal expression. The first discovery of the novel lncRNA TCONS_00072962 enriched the lncRNA expression profile in the SCA3/MJD mouse model.

## 1. Introduction

PolyQ diseases is a group of disorders caused by CAG repeat expansions within the, respectively, responsible genes, including Huntington disease (HD), dentatorubral-pallidoluysian atrophy (DRPLA), spinocerebellar ataxias (SCA1, SCA2, SCA3/Machado-Joseph disease, SCA6, SCA7, and SCA17) [1–3], and the recently discovered Huntington disease-like 2 (HDL2) [4, 5]. Among these, the SCA3/MJD is an autosomal dominantly inherited disorder with high clinical heterogeneity, such as progressive cerebellar ataxia, dysarthria, pyramidal signs, external ophthalmoplegia, dysphagia, and distal muscle atrophy, with wide range of age of onset (AO) from 4 to 75 years old [6, 7].

The SCA3/MJD, the most common case, accounts for 62.64% of autosomal dominant spinocerebellar ataxia in China [8]. The abnormal expansion of CAG in the causative gene ATXN3 coding region causes SCA3/MJD. Healthy individuals usually have 12–40 CAG repeats, while SCA3/MJD patients over 51 repeats [9, 10]. The abnormally translated polyQ tract leads to a conformational change in ATXN3, resulting in alternations of protein properties, including stability, subcellular location, and easier aggregation [11]. These alternations further lead to loss or gain of function and cause pathogenic effects. To explain the toxic effects, several hypotheses of pathogenic mechanisms, not mutually exclusive, have been presented, including aggregate formation [1, 11–13], disturbance of cellular protein and Ca2+ homeostasis [13–15], dysregulation of transcription [15, 16], axonal transport deficits [17, 18], impairment of mitochondrial function [15, 19, 20], and abnormal neuronal signalling [11].

Long noncoding RNA (lncRNA) is defined the nontranslatable RNA with the length of 200 nucleotides or above. The lncRNAs used to be regarded as the transcriptional “noise,” the products of RNA polymerase II transcription, and did not have the biological function. However, the emerging evidence has proved their significant roles in the regulation of gene transcription, posttranscriptional regulation, and epigenetic regulation [21, 22]. Previous studies suggested that lncRNAs regulate the gene expression and transcriptional processes by several different functional mechanisms. Some show function as transcriptional regulation in cis or trains, some as an organization of nuclear domains, and others as regulation of proteins or RNA molecules. All the evidence indicated that lncRNAs have great potential to impact physiological and pathological processes. Furthermore, it has been found that some transcripts of lncRNA encode small proteins [23], making the noncoding inappropriate any longer to name this class of RNA.

In recent years, accumulating studies have found that lncRNAs are associated with neurodegenerative diseases. Spinocerebellar ataxia type 8 (SCA8), a kind of slowly progressive ataxia, is caused by the abnormal expansion of (CTG)n within the responsible gene ATXN8. A study proposes that the pathogenesis of SCA8 involves both protein and RNA gain-of-function mechanisms. (CTG)n-expanded ATXN8 encodes a pathogenic protein, and the antisense strand encodes CUG-enriched lncRNA ATXN8OS which is deposited in the nucleus and activates alternative splicing, resulting in an alternation of the expression of GABA-A transport factor 4 (GAT4/Gabt4) and finally loss of the GABAergic inhibition [24].

In a separate study, the expression of lncRNA was compared between the normal brain tissue and the brain tissue of patients with Huntington disease. A total of 35 upregulated and 146 downregulated lncRNA molecules were identified, and NEAT1 was selected by Bioinformatics. Based on the cell-level experiments, it was found that overexpression of NEAT1 was significantly resistant to H_2_O_2_-induced cellular damage, providing a new potential strategy for clinical treatment of the Huntington's disease [25].

To further explore the pathogenesis of SC3/MJD at RNA level, the lncRNAs specifically expressed in SCA3/MJD mice were investigated in this study.

## 2. Materials and Methods

### 2.1. SCA3/MJD Mice

SCA3/MJD mouse model (B6; CBA-Tg (ATXN3^∗^) 84.2Cce/IbezJ; ID: 012075) from Jackson Laboratory was used, and the second generation was used in this study. The CAG repeats in the first generation mice are 84, and the *ATXN3* gene is widely expressed in various organs of the body, including the cerebellum, cerebral cortex, heart, lung, spleen, liver, and skeletal muscle [26, 27]. The SCA3/MJD adult mice (32 weeks old) of the second generation, in which carrying *ATXN3* positive rate is about 50%, and comparable age, number, and weight wild-type mice were used for experimental analysis. The study was approved by the Ethics Committee in Xiangya Hospital of Central South University.

### 2.2. Validation of Genotype of SCA3/MJD Mice

Validation of genotype was conducted in the second generation. Polymerase chain reaction (PCR), agarose gel electrophoresis, and capillary electrophoresis sequencing were used for genotype validation. Genomic DNA was extracted from mice tails. CAG repeats were amplified using a pair of primers 5′-CCAGTGACTACTTTGATTCG-3′ (forward) 5′-TGGCCTTTCACATGGATGTGAA-3′ (reverse). The amplification reactions contained 1 *μ*L genomic DNA (50 ng/*μ*L), 0.2 *μ*L rTaq DNA polymerase (Takara, Japan), 0.2 *μ*L dNTPs, 0.2 *μ*L of each primer (100 ng/*μ*L), 7.2 *μ*L sterile water, and 1.0 *μ*L 10x buffer (TaKaRa, Japan), for a total of 10 *μ*L. The amplification was performed in Mastercyclers (Eppendorf AG, 22331 Hamburg, Germany) under the following conditions: initial denaturation at 95.0°C for 5 minutes, followed by 38 cycles of 95.0°C for 30 seconds, 59.0°C for 30 seconds, and 72.0°C for 30 seconds. PCR products were detected by 1% agarose gel electrophoresis (120 v, 30 min), and the results of PCR amplification were observed on imaging system after 15 minutes of ethidium bromide (EB) staining. Capillary electrophoresis sequencing was used for testing the repeats number of (CAG)n and performed on ABI 3730XL DNA Analyzer (Applied Biosystems, Foster City, CA, USA).

### 2.3. Validation of Phenotype of SCA3/MJD Mice

The phenotype was validated using the footprint and rotating tests. For footprint pattern analysis, the hind paws of mice were painted with black ink and the forepaws were painted with red ink. The mice walked along a narrow corridor paved with white paper. Pretraining was conducted for one week before the formal test. Mice were tested three times with 5-minute intervals. Stride length, hind paw width, front paw width, and front/hind footprint overlap were measured. For rotation, mice were placed on a rotating rod and must maintain its balance. The interval from the start of the rod rotating to the mice falling from the rotating rod was recorded. Mice were tested on separate trials at fixed speeds including 10 r/min and 20 r/min.

### 2.4. lncRNA-Seq

lncRNA-Seq, a high-throughput sequencing, was performed in BGI. After extracting the total RNA from mice cerebellum (three SCA3/MJD mice versus three wild-type mice), mRNA and noncoding RNAs are enriched by removing rRNA from the total RNA. By using the fragmentation buffer, the mRNAs and noncoding RNAs are fragmented into short fragments (about 200~500 nt), then the first-strand cDNA is synthesized by random hexamer-primer using the fragments as templates, and dTTP is substituted by dUTP during the synthesis of the second strand. Short fragments are purified and resolved with EB buffer for end reparation and single nucleotide A (adenine) addition. After that, the short fragments are connected with adapters, and then the second strand is degraded using UNG (uracil-N-glycosylase) finally [28]. After agarose gel electrophoresis, the suitable fragments are selected for the PCR amplification as templates. During the QC steps, Agilent 2100 Bioanalyzer and ABI StepOnePlus real-time PCR system are used in quantification and qualification of the sample library. At last, the library could be sequenced using Illumina HiSeqTM 2000 or other sequencers when necessary. Differential expression analysis for both predicted novel lncRNA and lncRNA from the database has proceeded. It was compared between SCA3/MJD and wild-type mice (one by one, grouped randomly) through the Cuffdiff software to calculate the FPKM value of the gene or transcript in both samples and to detect the presence of differential expression. Also, group differential expression analysis was also performed by NOIseq method.

### 2.5. Quantitative Real-Time PCR

The total RNA from mice cerebellum (six SCA3/MJD mice versus six wild-type mice) was reversely transcribed to cDNA with a kit (Thermo Scientific, RevertAid First Strand cDNA #K1622). The 44 most differentially expressed lncRNAs were screened for further validation by qRT-PCR assays (Maxima SYBR Green qPCR Master Mix, CFX96, Bio-Rad, USA). *β*-Actin was used as an internal reference in the qRT-PCR analyses. The primers (see Table S1 in the Supplementary Material) were designed using Primer3 (http://bioinfo.ut.ee/primer3-0.4.0/). qRT-PCR assay was performed in triplicate in a volume of 20 *μ*L containing 1 *μ*L of cDNA. The relative expression level of lncRNA was calculated using the 2^−ΔΔCt^ method and Ct > 35 were excluded. A Wilcoxon rank sum test was used for statistical analyses, and *p* < 0.05 was considered statistical significance.

### 2.6. Bioinformatics Analysis

We conducted some biological analysis of the differentially expressed lncRNAs by search and comparison of databases such as NONCODE v5 (http://www.noncode.org/index.php), FANTOM5 (http://fantom.gsc.riken.jp/5/), STRING v.10.0 (http://version10.string-db.org/), and Gene Ontology Consortium (http://www.geneontology.org/) to predict the location, distribution, and function of these lncRNAs.

## 3. Results

### 3.1. Genotyping and Phenotyping of the SCA3/MJD Mice

The SCA3/MJD mice that carry the ATXN3 were genotyped and phenotyped. As a result, the ATXN3 carrying rate was about 50% in the second generation of SCA3/MJD mice, by Jackson Laboratory's report. The number of CAG repeats was 84 or 83 in the second generation SCA3/MJD mice. In rotation test, the interval of mice keeping balance on the rotating rod was significantly different between the SCA3/MJD and wild-type mice no matter the rotation speed was 10 r/min or 20 r/min (see Table 1), indicating that the balance and motor abilities of the SCA3/MJD mice were much worse relative to the control mice. Morphologically, wider hind paw width was evident in SCA3/MJD mice.

Abnormal gait was observed by analyzing the footprint pattern. In contrast to the control mice's straight line with regular alternating gait, the SCA3/MJD mice showed unstable movements in a way that weaved from side to side when walked along the narrow corridor. Altogether suggest that the SCA3/MJD mice have similar clinical manifestations with the SCA3/MJD patients.

### 3.2. lncRNA-Seq

By using RNA-seq, 10,443 of novel and 13,395 of known lncRNAs were detected in 3 of the SCA3/MJD and 3 of the WT mice. One by one differential expression analysis showed total 2964 upregulated and 4376 downregulated lncRNAs, respectively, in the three experimental groups. More specifically, there were 745, 776, and 1250 of the upregulated lncRNAs in three groups, in contrast to the significantly increased numbers of 1285, 1065, and 1600, the downregulated lncRNAs, respectively, in three groups. Further group differential expression analysis found 193 of the upregulated and 467 of the downregulated lncRNAs in the SCA3/MJD mice. The lncRNAs with differential expression identified by the one by one differential expression analysis in two or three groups were chosen for further validation. The lncRNAs were sorted based on their FPKM and *p* values in the group differential expression analysis. For both differential expression analyses, a total of 44 lncRNAs were chosen for further experiments because they were statistically significant.

### 3.3. qRT-PCR

**Figure 1:**
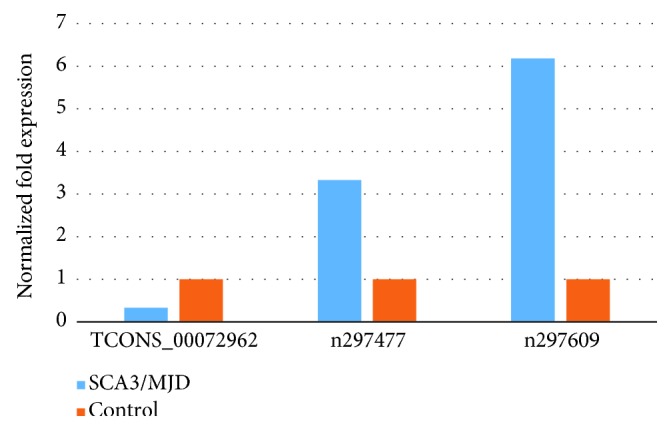
The expression level of the three lncRNAs.The expression level of three lncRNAs was different between wild-type and SCA3/MJD mice with statistical significance. The two known lncRNAs, n297477 and n297609, were upregulated in the cerebellum of SCA3/MJD mice. The expression level was increased by 3.329 times (*p* = 0.016) and 6.182 times (*p* = 0.041), respectively. The novel lncRNA TCONS_00072962 was downregulated (*p* = 0.036) in the cerebellum of SCA3/MJD mice, which was nearly one-third of that in wild-type mice.

lncRNA number TCONS_00031478 was excluded due to Ct > 35. In the rest 43 of the chosen lncRNAs, 3 of the lncRNAs were validated to be differential expression between SCA3/MJD and WT mice cerebellum. It turned out that two of the three are the known lncRNAs number n297477 (*p* = 0.016) and number n297609 (*p* = 0.041) (NONCODE v5.0: http://www.noncode.org/), and the remaining one belonged to a novel lncRNA (number TCONS_00072962) (*p* = 0.036). 2^−△△Ct^ method was used for calculating the relative expression level of each lncRNA. When compared with wild-type mice, the two known lncRNAs, n297477 and n297609, were upregulated by 3.329-fold and 6.182-fold, respectively, in the cerebellum of SCA3/MJD mice (see Figure 1), while the expression level of novel lncRNA TCONS_00072962 was downregulated in the cerebellum of SCA3/MJD mice, which was nearly one-third of that in control mice.

## 4. Discussion

In this study, 2964 upregulated lncRNAs and 4376 downregulated lncRNAs were identified specifically in the SCA3/MJD mice using lncRNA-Seq analysis. Additionally, differentially expressed three lncRNAs, including one novel lncRNA and two known lncRNAs, were further characterized.

The lncRNA n297477 is transcribed from the antisense strand of the chr11: 6270375–6271530, which is highly expressed in mouse heart, hippocampus, liver, lung, spleen, and thymus. According to the records in database FANTOM5, n297477 is considered one of the transcripts of the *TMED4* gene whose promoter is located in the sense strand at position 142–392 (chr11: 6270517–6270767) and TATA box starts at 377 (chr11: 6270712) (http://www-bimas.cit.nih.gov/molbio/proscan/). According to the database STRING v.10.0, Tmed4 and ubiquitin C may be functional partners to each other, whereas the ubiquitin C might be a functional partner of ataxin3 encoded by the SCA3/MJD pathogenic gene *ATXN3* (Table 2; http://version10.string-db.org/cgi/network.pl?all_channels_on=1&block_structure_pics_in_bubbles=0&direct_neighbor=1&hide_disconnected_nodes=0&hide_node_labels=0&network_display_mode=svg&network_flavor=evidence&targetmode=proteins&identifier=9606.ENSP00000376965; http://version10.string-db.org/cgi/network.pl?all_channels_on=1&block_structure_pics_in_bubbles=0&direct_neighbor=1&hide_disconnected_nodes=0&hide_node_labels=0&network_display_mode=svg&network_flavor=evidence&targetmode=proteins&identifier=9606.ENSP00000404042).

Given that the ubiquitin-proteasome system (UPS) is involved in the pathogenesis of SCA3/MJD [29], it is plausible to speculate that lncRNA may participate in gene expression regulation when it is located close to the transcription start site of the promoter region. Since the lncRNA n297477 and TMED4 gene starting site are separated by 16 bp in the middle, and the gene promoter region is located in the lncRNA coding region, it is highly possible that the n297477 participates in TMED4 expression regulation. We assume that the altered expression of the TMED4 affects its interaction with UBC, which affects the efficiency of ubiquitin C in the ubiquitin-proteasome system. Thus, the lncRNA n297477 may be involved in the regulation of ataxin-3 protein degradation by regulating UPS. The elevated expression of n297477 in the SCA3/MJD mice may serve as a response to the abnormal degradation of toxic ataxin-3.

The lncRNA n297609 is transcribed from the antisense strand of the chr9: 44047488–43856016, which is highly expressed in mouse heart, hippocampus, liver, lung, spleen, and thymus. According to the records in database FANTOM5, n297609 is considered one of the transcripts of the *THY1* gene which is highly homologous to humans. The lncRNA n297609 mainly participates in the protein phosphorylation (GO: 0006468), cell adhesion (GO: 0007155), cellular response to heat (GO: 0034605), positive regulation of transcription (GO: 0045893), and negative regulation of cell migration (GO: 0030336). Previous studies pointed out that protein casein kinase 2- (CK2-) dependent phosphorylation can control the stability, nuclear localization, and aggregation of ataxin-3 [30]. Thus, it is logical to assume that the lncRNA n297609 may be involved in biological processes, such as protein phosphorylation, and protein casein kinase 2- (CK2-) dependent phosphorylation plays a crucial role in SCA3/MJD pathophysiology [30]. However, this assumption requires further validation.

Moreover, we also found a novel lncRNA TCONS_00072962 with the genomic location at chr7:119737479–119737966, contributing to the expansion of lncRNA expression profiles of the mouse.

## 5. Conclusions

In summary, we used lncRNA-Seq to profile cerebellar expression in SCA3/MJD mice and identified three potential lncRNAs significantly associated with the disease. These identified lncRNAs will be beneficial for the further understanding of cerebellum gene coexpression network correlating with disease progression. Furthermore, investigation on the lncRNA-associated neuroprotective factors remains to be necessary for the elucidation of the therapeutic target implication.

## Figures and Tables

**Table 1:** The results of rotation test.

Rotation speed	Time (seconds)	Wild-type mice (*n* = 6)	SCA3/MJD mice (*n* = 6)	*p* value
10 r/min	Mean ± SD	250.067 ± 9.487	104.400 ± 8.902	0.023
20 r/min	Mean ± SD	196.467 ± 8.126	34.600 ± 6.710	0.002

The wild-type mice performed much better than the SCA3/MJD mice in the rotation test.

**Table 2:** The summary of genetic association.

Gene name	Associated gene name	Proteins of associated gene
ATXN3	VCP	Valosin containing protein
	UBC	Ubiquitin C
	KCTD10	Potassium channel tetramerisation domain containing 10
	RAD23A	RAD23 homolog A
	RAD23B	RAD23 homolog B
	PARK2	Parkinson protein 2
	USP13	Ubiquitin specific peptidase 13
	UBE4B	Ubiquitination factor E4B
	STUB1	STIP1 homology and U-box containing protein 1
	SERPINC1	Serpin peptidase inhibitor, clade C (antithrombin), member 1

UBC	PSMD4	Proteasome (prosome, macropain) 26S subunit, non-ATPase 4
	HSP90AA1	Heat shock protein 90 kDa alpha (cytosolic), class A member 1
	HGS	Hepatocyte growth factor-regulated tyrosine kinase substrate
	PSMC2	Proteasome (prosome, macropain) 26S subunit, ATPase 2
	TSG101	Tumor susceptibility gene 101
	UBE2D2	Ubiquitin-conjugating enzyme E2D 2
	PSMD14	Proteasome (prosome, macropain) 26S subunit, non-ATPase 14
	CUL1	Cullin 1
	RPS27A	Ribosomal protein S27a (156 aa)

## Data Availability

The location of all lncRNAs is from the mm10 database.

## Supplementary Materials

Supplementary Materials Table S1: the supplementary material included 44 lncRNA locations, dysregulation (upregulated/downregulated), and primers used for qPCR amplification.
