# Supplementary material for: Cerebellar lncRNA Expression Profile Analysis of SCA3/MJD Mice
Source: Int J Genomics. 2018 Jun 25;2018:5383517. doi: 10.1155/2018/5383517 (PMC6036799; doi:10.1155/2018/5383517)
Supplement: Supplementary Materials — Table S1: the supplementary material included 44 lncRNA locations, dysregulation (upregulated/downregulated), and primers used for qPCR amplification. [file 5383517.f1.docx]

Table S1. The locus, dysregulation, and primers of novel and known lncRNAs

| lncRNAs | Locus | Dysregulation | Primers |
| --- | --- | --- | --- |
| TCONS_00022646 | chr13:104710801-104711126 | Down | F: GGAAAGAGGACGGAGGAGA  R: CGGGTATTGTCTCGAGTGC |
| TCONS_00029336 | chr18:46596227-46596669 | Down | F: GGAGGGACTTGGCTTTCTCT  R: AAGATGGACAGGGCAGGAT |
| TCONS_00031401 | chr16:4213622-4214287 | Down | F: TCAGGCTCTGGAAAGAGAAGA  R: GAGTAGATCGCGCTCGAAG |
| TCONS_00031478 | chr16:8738774-8739222 | Down | F: GCTGCCTCACAGAGGAGACC  R: GAGCGGAGCGAACGAGAC |
| TCONS_00050129 | chr3:32295909-32296386 | Down | F: TCCTCAGCTCTCACCCTCCT  R: TTGGTCTCTCTTTCCGCTCA |
| TCONS_00051774 | chr3:126899571-126903089 | Down | F: GGGCAGCTTTCTGATACAGG  R: ATTCTGCTCGCCATGATGTT |
| TCONS_00061874 | chr5:115636144-115638620 | Down | F: CTCTCTGCTCCAGTCGCTCT  R: AACGCACATACAAACCACCA |
| TCONS_00072962 | chr7:126880993-126882648 | Down | F: GCAGCAGATTCCACTACAATGA  R: CTTGCCTGTCACCACCATC |
| TCONS_00032213 | chr16:49912901-49913618 | Down | F: CTGATGGGACGAGAACTGG  R: CTGTATGAAGGATGGCAGCA |
| TCONS_00040993 | chr19:57372070-57375878 | Down | F: CTCTTCTGTGGGACGGTTCT  R: TCTTCCTGCCTTAGCCTCTG |
| TCONS_00041161 | chr19:4994341-4998258 | Down | F: ATGCCCTTTCAGCCTTCTTT  R: TCACCTTTGTCTGCCATCAG |
| TCONS_00051773 | chr3:126895798-126899430 | Down | F: CATCGGACTTGACCACACAG  R: GTTTGCCTTCTGCCATCTTC |
| TCONS_00051825 | chr6:92033069-92033475 | Down | F: TGCCCTCAACCCACAAGTAT  R: CTCAATCCCAGCCCTTATCA |
| TCONS_00057506 | chr4:15834344-15835221 | Down | F: TGCCAAGAATCCAGAAACC  R: CCAGAAACAGAATAGCACCAA |
| TCONS_00059106 | chr4:125166576-125168039 | Down | F: GCGTGGAAGGTAGGACTTGA  R: TGCGGAAGAGGACTGAGAAC |
| TCONS_00070010 | chr6:117962919-117967435 | Down | F: GGTGAGACAGCGGAGTAAGC  R: TCAGAGGTGAAGGGATTTGC |
| TCONS_00072160 | chr7:88136342-88138469 | Down | F: GAGTTCTTGCCCTGGAGTCA  R: GAGAGTGGAATGGAGGTGGA |
| TCONS_00077524 | chr8:58607127-58608012 | Up | F: AAGGAGTATCTTATCTCTGTGAGGA  R: CTGAAACAATGGAAGCAGGT |
| TCONS_00078970 | chr8:129117567-129117934 | Down | F: CTGCACAGCACCCACTGC  R: CGTGCAGAGCTGTCGAAGAA |
| TCONS_00080843 | chr8:90483575-90484673 | Down | F: CTCCTCACATCCTCCTGCTG  R: CGCTCCTGTCTTCTTTGTGG |
| TCONS_00010060 | chr10:129849325-129851166 | Down | F: TCCTGCCTTGATCTTGCTCT  R: TCCAGTCCAGCTCTTCCACT |
| TCONS_00025532 | chr14:64465699-64468462 | Down | F: TCCTTTGTGGGTTTGTTGCT  R: TCTTTGCCTCCTGTATTTCCA |
| TCONS_00035057 | chr17:8966714-8969175 | Down | F: GGCTGATAGGAAGGGAGCA  R: CAGAAGGGTTCAAGGGTGTC |
| TCONS_00051814 | chr3:126994074-126999391 | Down | F: CACCACAAATCAGAGGCAGA  R: CACACCACCAGCCAACATAC |
| TCONS_00051817 | chr3:127003787-127006361 | Down | F: CAGGGCGACAAAGTGAGTTC  R: CTGAGAAGGCAGCGGATTAG |
| TCONS_00062154 | chr5:124800979-124804895 | Down | F: ACACACAGCGACCATAACCA  R: ACCCTCTCCTCCCAGACATT |
| TCONS_00081372 | chr8:122752619-122755736 | Down | F: TGCTGAACCAATACCACCAC  R: AACGGCAGGAGTAACAAAGG |
| n271202 | chr9:44286860-44291886 | Up | F: TGTGTCGTTCCTCCTGTCTG  R: CATCCACCCAGAAACATTCA |
| n287116 | chr15:58007439-58010345 | Down | F: TGGACTCTGGCATTTCTGC  R: TTCAACCCTCTTGCTGACCT |
| n294100 | chr3:40594964-40596566 | Up | F: TCCCTTCCTCCCTCCTTTC  R: GTTCCAAGACACCAGGCAAT |
| n295268 | chr17:34738043-34740535 | Up | F: TATTCCTGTTCCTCGCCATC  R: CAGCAGACAGAGATGGGTCA |
| n297477 | chr11:6170378-6174853 | Up | F: GCTGCTGGTGCTTACTGTGT  R: ATGGTCTCGTCGGGTATTTC |
| n281660 | chr4:33332397-33336061 | Up | F: AGGCAAGACACAAACAAGCAG  R: AGGGAGGTGAGGCAGAAGTC |
| n291706 | chr4:131907847-131909601 | Up | F: CGTTGGAATGTCTGGTTAGC  R: TGAGGCTGGCTTAGTGCTT |
| n294215 | chr4:108452662-108453902 | Up | F: TCGGTTCATTTCTCGTGGA  R: ATCTGCTTTGGGAAGGGATT |
| n297214 | chr1:173288535-173290929 | Down | F: AGTGCCTCCCTGCTGTTCT  R: GGACTTGCCTTTGGAATGAC |
| n344293 | chr11:63892990-63896761 | Down | F: GCACCCTTTCCCATCCTTAC  R: CCTGCCACTCTCCCATTCTA |
| n413384 | chr2:25430935-25435619 | Up | F: GCTGAGAGAGAAGCCAAAGC  R: GGTCACACCCTCCACAAAGT |
| n414326 | chr11:69153957-69156149 | Down | F: CCTTCTTCTTTCCTCCAGTGC  R: CCACCTGCTTTCTGCCATAG |
| n5654 | chr10:39978272-39978485 | Up | F: CTGTGTAGAGCACCCGAAAC  R: TCAATGGCAGATAGCAGTTG |
| n413527 | chr3:88003026-88007403 | Up | F: AGGAAGGACAACGCAGAAGA  R: GGAGGATGGCAGAACAGAGT |
| n272743 | chr4:12908837-12911361 | Down | F: CGCTGCCTTGTCTTCTTAGC  R: TCTGTAGGGCTGGGTTCATC |
| n4341 | chr4:43505532-43505933 | Up | F: CACTGCCTGCGTCACTATGT  R: ATGTTCCTTATCCTTTCGCCTA |
| n297609 | chr9:43851466-43856670 | Up | F: TAAGCCCTCATCCTCATTGG  R: GGAAGTTGGACTGTGCCTGT |
| β-actin |  |  | F: TGAAGGTGACAGCAGTCGGTTG  R: GGCTTTTAGGATGGCAAGGGAC |

F: represents forward primer; R: represents reverse primer

Novel lncRNAs: using the ‘TCONS’; known lncRNAs: using the ‘n’

The locus of all lncRNAs is from the mm9 database（Based on raw sequencing data）.
